# Immobilization of Colloidal Monolayers at Fluid–Fluid Interfaces

**DOI:** 10.3390/gels2030019

**Published:** 2016-07-08

**Authors:** Peter T. Bähler, Michele Zanini, Giulia Morgese, Edmondo M. Benetti, Lucio Isa

**Affiliations:** 1 Laboratory for Interfaces, Soft matter and Assembly, Department of Materials, ETH Zurich, Vladimir-Prleog-Weg 5, 8093 Zurich, Switzerland; baehler.p@gmx.ch (P.T.B.); michele.zanini@mat.ethz.ch (M.Z.); 2 Laboratory for Surface Science and Technology, Department of Materials, ETH Zurich, Vladimir-Prleog-Weg 5, 8093 Zurich, Switzerland; giulia.morgese@mat.ethz.ch (G.M.); edmondo.benetti@mat.ethz.ch (E.M.B.)

**Keywords:** colloids, fluid interfaces, immobilization

## Abstract

Monolayers of colloidal particles trapped at an interface between two immiscible fluids play a pivotal role in many applications and act as essential models in fundamental studies. One of the main advantages of these systems is that non-close packed monolayers with tunable inter-particle spacing can be formed, as required, for instance, in surface patterning and sensing applications. At the same time, the immobilization of particles locked into desired structures to be transferred to solid substrates remains challenging. Here, we describe three different strategies to immobilize monolayers of polystyrene microparticles at water–decane interfaces. The first route is based on the leaking of polystyrene oligomers from the particles themselves, which leads to the formation of a rigid interfacial film. The other two rely on in situ interfacial polymerization routes that embed the particles into a polymer membrane. By tracking the motion of the colloids at the interface, we can follow in real-time the formation of the polymer membranes and we interestingly find that the onset of the polymerization reaction is accompanied by an increase in particle mobility determined by Marangoni flows at the interface. These results pave the way for future developments in the realization of thin tailored composite polymer-particle membranes.

## 1. Introduction

Monolayers of colloidal particles are widely used both as model systems [1,2,3] as well as in a large range of applications, spanning from the fabrication of lithography masks [4,5] and anti-reflective coatings [6] to emulsion stabilizers [7] and patterning elements for ultra-thin polymeric membranes [8]. Standard protocols to prepare monolayers of colloidal particles adsorbed onto a solid substrate involve direct adsorption from solution (including sedimentation [3] and electrostatic adsorption [9,10]), spin-coating [11], controlled drying [12], convective assembly [13] and electric-field-assisted deposition [14,15]. In all of these cases, dense particle monolayers are produced, mostly with particles sharing contacts in closely packed arrays. Applications, e.g., in biosensing [16] and patterning [5,17,18], and fundamental studies, e.g., in tailoring surface adhesion [19] and friction [20] by particle adsorption, often also require non-close-packed monolayers with control on both the particle size and the spacing between them. This can be achieved by first depositing a close-packed monolayer and then by reducing the particle size by reactive etching [21], or alternatively by depositing the particles onto stretchable elastomers [22]. Albeit successful and easy to implement, these methods present some limitations. The former process does not afford independent control on size and spacing, i.e., the two are linked by the initial particle diameter. Moreover, sizes can only be reduced up to a given point before the particles lose their shape integrity. The latter strategy involves high-temperature steps, which restrict its applicability to inorganic particles. Another alternative exploits the self-assembly of colloids at fluid–fluid interfaces [4,23]. In this case, monolayers of particles are first formed at the interface between two fluids, i.e., oil-water or air-water, and are then deposited onto a solid substrate. The presence of long-range electrostatic forces between particles of suitable surface charge and wettability enables the possibility to obtain regular, non-close-packed 2D lattices [24,25]. These forces are dipolar in origin and can either be generated by charges on the particle–water surface or on the particle–nonpolar fluid (air or oil) surface, depending on the materials of interest. In particular, in the case of dominant charges on the particle-nonpolar fluid surface, it has been shown that adsorption of ions from air in atmospheric conditions, or migration of ions from the water side, can greatly reduce the effective surface charge density and thus lead to weaker repulsion [26,27]. These contributions are less important in the case of highly water-insoluble oils, such as purified alkanes, which tend to maintain higher unscreened surface charges leading to stronger electrostatic repulsion. For the purpose of structural tailoring, spacings up to ten particle diameters can be achieved and smoothly tuned by controlling the number of particles per unit area injected at the interface [4] or by monolayer compression [26,28]. The most complicated part of the process is the transfer of the particles from the interface to the substrate without destroying the monolayer’s integrity. Different approaches have been devised, either using solvent exchange [23] or by choosing oil phases with suitable viscosities and volatilities [4]. The process remains nevertheless challenging due to limited adhesion between the particles and the substrate. Additional routes have been pursued by using soft deformable particles that show an increased area of contact and thus higher adhesion with the template, but, in this case, the range of available inter-particle spacing is limited and it is dictated by the range of soft steric interactions at the interface [29].

In this paper, we describe three different approaches that can be used to immobilize particle monolayers directly at a fluid–fluid interface before deposition, which could therefore be used in the future to circumvent the issues described above. The paper is organized as follows. We initially present a first route, where immobile monolayers of polystyrene (PS) particles are created spontaneously at a water–decane interface without the addition of any external ingredient. The kinetics of the immobilization process is followed by in situ microscopy and particle tracking. We then move to another approach, where two different interfacial polymerization routes are followed to immobilize the particles at the water–decane interface into thin polymer membranes. The presentation and discussion of the experimental results is followed by conclusions and a perspective for future work. An experimental section detailing the materials and methods used in our work closes the manuscript.

## 2. Results and Discussion

**Figure 1:**
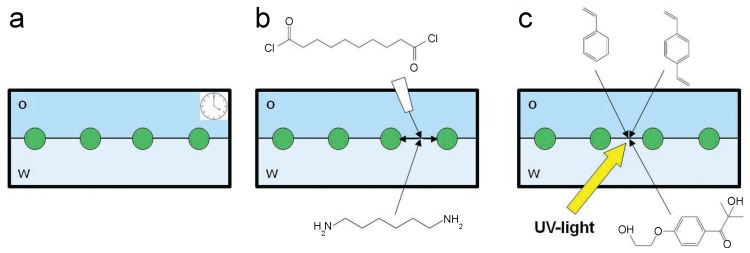
Schematics of the three different immobilization routes. (**a**) spontaneous immobilization at a water–decane interface. Particles within the monolayer simply become immobile with time; (**b**) nylon interfacial polymerization. After the spontaneous interfacial adsorption of 1,6-diaminohexane from the water phase, sebacoyl dichloride is injected into the organic phase to start the polymerization; (**c**) polystyrene interfacial polymerization. After the spontaneous interfacial adsorption of the monomer (styrene) and crosslinker (*p*-divinylbenzene) from the oil phase and of the initiator (Irgacure 2959) from the water phase, the system is illuminated by UV light to initiate the free radical polymerization of styrene at the interface.

Figure 1 illustrates schematically the three different approaches that we have investigated to study PS particle monolayer immobilization at the water–decane interface. Results and discussion follow for each route.

### 2.1. Spontaneous Immobilization

Experiments aimed at studying particle dynamics within a monolayer at a water–decane interface revealed that particle mobility was a strong function of the residence time of the particles at the interface. Systematic investigations showed that, given a sufficient amount of time, the particles became completely immobilized without distorting the interface microstructure. The experiments were carried out as follows (additional details in the Experimental Section). A given number of fluorescent polystyrene particles of either 2.8 or 1.08 *μ*m diameter was injected using a micropipette at a macroscopically flat oil-water interface created in a customized sample cell. A solution of 50:50 ultra-pure water and isopropanol was used to aid the spreading of the particles at the interface [30]. The latter was obtained by filling a metallic ring to its rim with water and carefully covering it with decane, after which the sample cell was sealed at the top with a glass slide. The cell was then placed under a microscope, where videos of the particles at the interface were captured by fluorescence microscopy as a function of waiting/residence time at the interface. The videos were successively processed to extract particle trajectories and to compute the particle mean squared displacements (MSD) as a function of time. The MSD is a standard quantity to describe the modality of the motion of colloidal particles driven by thermal fluctuations. In the case of freely diffusing particles, the MSD plotted as a function of time shows a slope of 1 in a log-log plot. Slopes below 1 indicate sub-diffusive behavior characteristic of crowded or super-cooled systems, and flat lines indicate complete local caging of the particles that cannot move beyond a specific distance, as in the case of completely immobilized particles.

**Figure 2:**
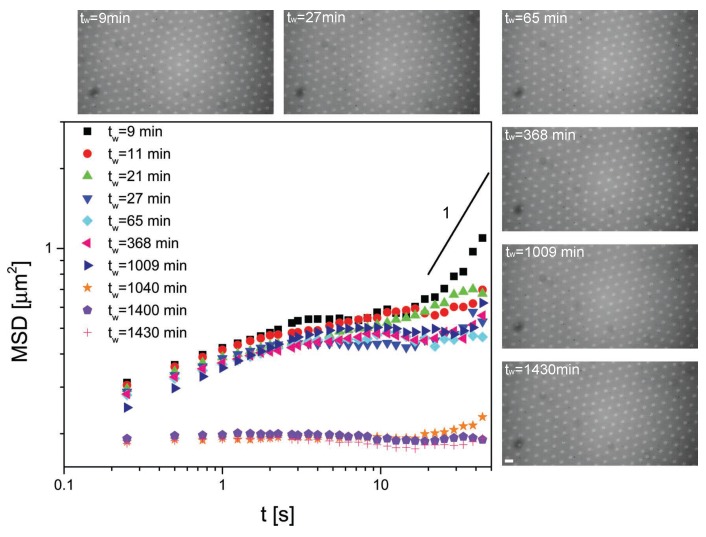
Mean squared displacements (MSD) versus time for 2.8 *μ*m polystyrene (PS) particles at the water–decane interface as a function of residence time at the interface $t_{w}$. A solid line with slope 1 representative of freely diffusive motion is included for comparison. The micrographs show the interface microstructure at various waiting times. The scale bar is 10 *μ*m.

**Figure 3:**
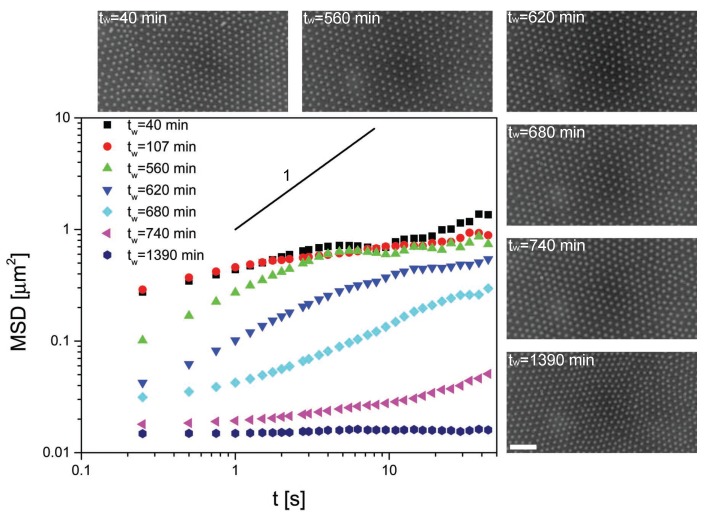
MSD versus time for 1.08 *μ*m PS particles at the water–decane interface as a function of residence time at the interface $t_{w}$. A solid line with slope one representative of freely diffusive motion is included for comparison. The micrographs show the interface microstructure at various waiting times. The scale bar is 10 *μ*m.

Figure 2 and Figure 3 show the MSDs of the 2.8 and 1.08 *μ*m diameter particles, respectively, for different residence times $t_{w}$ at the interface, where $t_{w}=0$ corresponds to the time at which the particles were injected at the interface. The data are accompanied by a series of representative microscopy images showing the interface microstructure at the various waiting times. Starting from the data in Figure 2, we describe the mobility and the arrangement of the PS particles at the interface. The first observation to be made is that the particles self-assembled into non-close-packed crystals due to electrostatic repulsion. The corresponding MSD at short waiting times displayed the expected behavior. At short times, the MSD increased sub-linearly with time, indicating sub-diffusive motion of the particles interacting with their neighbors. After a few seconds, the MSDs plateaued and became flat, indicating that the particles became completely caged by their neighbors. This situation corresponds to particles rattling around their lattice positions. Remarkably, the rattling happened over distances much smaller than the typical particle size or inter-particle distance, indicating strong local trapping. After a waiting time of approximately 16 h, the situation was radically different. Here, the local rattling of the particles in the lattice had completely disappeared and the MSD was flat at all times. The value of the MSD corresponded to displacements of the order of one tenth of the particle diameter, which basically constitutes the resolution limit of the particle tracking. We can then conclude that, after a few hours, the particles stopped moving completely and a fully immobile monolayer was formed. Very interestingly, no appreciable changes in the interface microstructure were found. The particle monolayer maintained its non-close-packed crystalline structure, as can be seen by the micrographs. Analogous results are seen in Figure 3 for the smaller PS particles.

**Figure 4:**
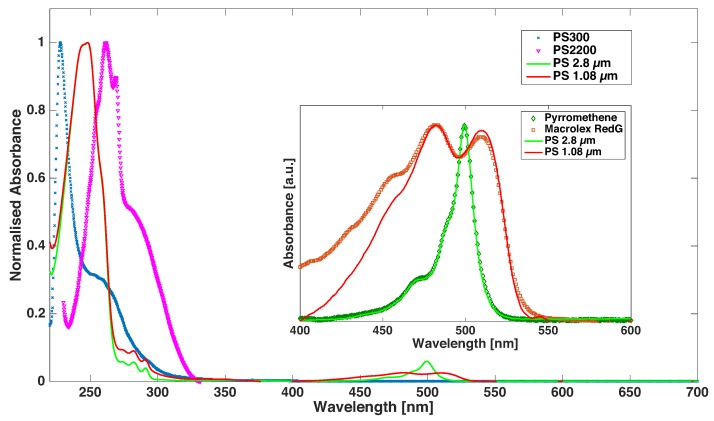
UV-Vis spectra of supernatants after overnight exposure to decane for the 2.8 (**green** solid line) and 1.08 *μ*m (**red** solid line) diameter PS particles. The spectra are combined with the UV-Vis spectra of reference decane solutions of pure PS (molecular weight 300 Da (**blue** crosses) and 2200 Da (**purple** triangles)) and of the red (Macrolex RedG (**red** squares)) and green (Pyrromethene (**green** diamonds)) dyes, zoomed in the inset for the relevant wavelength region.

The complete immobilization of the particles suggests that a strong elastic film was formed at the interface as a function of the particle residence time. This result was initially surprising, given that no other component was added to the system during the experiments. We therefore proceeded to unravel the reason for the formation of this very stiff membrane. After testing that this was not due to the presence of surface-active contaminations coming from the sample cells or the liquids used, the only conclusion left was that the particles themselves were responsible for their immobilization. This was presumably due to the appearance of surface-active impurities leaking from the particles into decane and then adsorbing at the interface, as we later confirmed by the following experiment. After drying 0.1 mL of the PS particles aqueous stock suspension at 40 ${}^{\circ }$C overnight in a vacuum oven in an Eppendorf tube, they were subsequently redispersed in 1.5 mL of purified decane, sonicated for at least 10 min and allowed to sediment overnight. We then took the supernatant and measured its interfacial tension against ultra-pure water. We measured a strong reduction of the interfacial tension from the pure water–decane value of 53 mN/m down to 32 mN/m (1.08 *μ*m) and 40 mN/m (2.8 *μ*m) after 3 h (significantly less than the timescales for interfacial arrest reported in Figure 2 and Figure 3), which confirmed the leaking of surface-active species from the PS particles into decane. We then proceeded to identify these surface-active species by running UV-Vis spectroscopy on the same supernatants. The results are shown in Figure 4. The spectra for the supernatants of the 1.08 and 2.8 *μ*m particles (red and green curves) showed distinctive bands in two regions. A first very intense band in the 250 nm range and a second less intense band in the 400–550 nm region. In order to identify and confirm the origin of these bands, we also ran UV-Vis spectroscopy on decane solutions of the same dye molecules used to render our PS particles fluorescent and on decane solutions of PS oligomers having two molecular weights, 300 and 2200 Da respectively. The first band for the particle supernatants was compatible with the peak of the PS oligomers, as shown by comparing the green and red curves in Figure 4 with the blue and purple ones obtained for the pure polystyrene oligomers. The fact that the peak position of the green and red curves was identical and lay in between the ones for PS300 and PS2200 indicates that oligomers of comparable molecular weight, between 300 and 2200 Da, were released into decane from both particles. These were most likely un-reacted and non-crosslinked PS chains left behind after particle synthesis. By comparing the peaks in the longer-wavelength region, we confirmed that dye molecules were also leaking out into decane. Identical spectral features were in fact found in the supernatant and in the pure dye solutions, as evidenced in the inset to Figure 4. In order to assess which one of the two substances leaking from the particles was responsible for the creation of the strong elastic film at the interface, we measured the surface tension reduction of a pure PS solution and of the dye solutions at the water–decane interface and compared it to the one measured for the particle supernatants. Pendant drop tensiometry showed that, albeit surface-active, the dye molecules showed a saturation of the water–decane interfacial tension reduction at a few mN/m below the pure water–decane level over several hours, and therefore cannot be responsible for the stronger interfacial tension reduction measured for the supernatants. Measurements on a PS300 solution (and we expect similar results for PS2200) instead showed a dramatic reduction of interfacial tension, which saturated at 20 mN/m for high PS concentrations already after a few minutes. We can therefore conclude with certainty that the formation of the strong interfacial film immobilizing the particles at the interface was due to interfacial adsorption of non-crosslinked PS oligomers leaking from the particles when exposed to decane.

### 2.2. Nylon Interfacial Polycondensation

The results presented in the previous section showed that it was possible to immobilize particles into stable regular lattices at the water–decane interface by solely waiting for a sufficient amount of time for the PS oligomers to build up an elastic monolayer. This approach, spontaneously occurring for our PS beads trapped at a water–decane interface, is very simple but time-consuming and only applicable to systems releasing surface-active species capable of creating highly elastic films. Despite being ”frozen” in place, the particles are only surrounded by a very thin polymer layer, whose composition and thickness cannot be externally controlled. This section and the next one describe two different possible routes to embed these regular particle arrangements into thicker and more controlled polymeric membranes.

The first route, schematically shown in Figure 1b, is based on the interfacial polycondensation reaction commonly known as the ”nylon rope trick”. In this case, one monomer, sebacoyldichloride, reacts with a second one, 1.6-diaminohexane, under the elimination of hydrogen chloride [31]. This reaction is particularly suited for our purpose since the two monomers can be dissolved into immiscible solvents, e.g., water and decane, and the reaction takes place only at the interface between these two fluids, where the monomers meet. In the presence of particles at the interface, these are entrapped by the nylon membrane as it grows.

**Figure 5:**
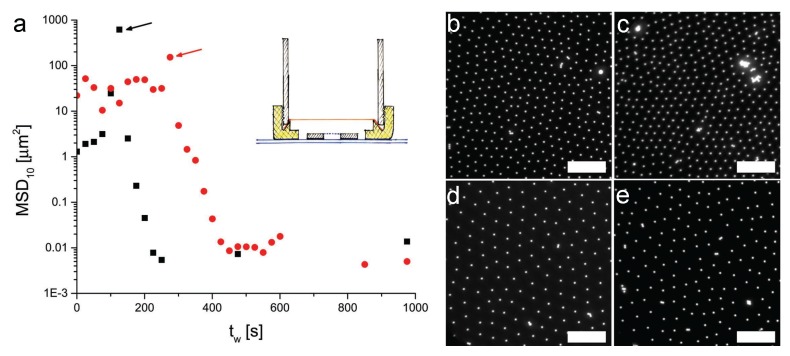
(**a**) MSD calculated after 10 seconds for 2.8 *μ*m PS particles at a water–decane interface during the interfacial polymerization of nylon plotted as a function of waiting time after injection of sebacoyldichloride, with (**red**) and without (**black**) filter paper. The arrows mark the points of highest particle mobility due to convective Marangoni flows at the interface. Inset: schematic of the sample cell used for the measurements. The horizontal dashed line represents the interface and the red line depicts the filter paper membrane; (**b**–**e**) fluorescence images of the particles at the interface before (**b** and **d**) and after polymerization (**c** and **e**), showing no qualitative difference in the microstructure. Images (**b**) and (**c**) are taken without the filter paper membrane; (**d**) and (**e**) with the filter paper membrane. The scale bars are 50 *μ*m.

We carried out these experiments by using a customized sample cell, as schematically depicted in the inset to Figure 5a. The cell consisted of a metallic ring with a sharpened edge, used to contain the water phase and pin the liquid interface, glued onto a microscope slide and enclosed by a cut-out centrifuge tube, whose cap was also glued onto the slide. The metallic ring was filled with a 6 mM water solution of 1.6-diaminohexane to achieve a flat water–air interface and then carefully covered by 0.5 mL of pure decane to create the water-oil interface. At these concentrations, the 1.6-diaminohexane rapidly formed an interfacial layer, as monitored separately by pendant drop tensiometry. A pipette tip, filled with the particles dispersed in the spreading solution previously described, was then brought in contact with the interface to produce a particle monolayer surrounded by 1.6-diaminohexane. At this stage, 100 *μ*L of a 34 mM sebacoyldichloride solution in decane were added to the oil phase and the dynamics of the particles at the interface was monitored as discussed above. Alternatively, a round piece of filter paper (MN 615, prewetted by decane) could be mounted above the interface and blocked by the screw-on cap of the centrifuge tube and additional oil (1–4 mL) could be inserted. The filter paper acted as a physical barrier to avoid flow at the interface caused by the injection of the additional decane containing the sebacoyldichloride. After a given amount of time, the second monomer reached the interface and the polymerization reaction started. The evolution of particle mobility was monitored as a function of time after the injection of the second monomer $t_{w}$ and it is shown in Figure 5a by plotting the MSD of the 2.8 *μ*m PS particles at ten seconds (MSD${}_{10}$) vs. $t_{w}$. The black square symbols refer to the case with no filter paper and the red circles to the case with filter paper. In both cases, the same qualitative behavior was observed. After an initial time where the MSD remained fairly constant, a sudden very large increase of particle mobility was seen (as highlighted by the arrows). Visual inspection showed that this corresponded to very violent convective flows at the interface, which then stopped fairly rapidly. After that, the particles became completely immobilized, with values of the MSD comparable to the ones reported in Figure 2. The strong convective flows were not due to mechanical disturbance of the interface upon injecting the second monomer, since they occurred after minutes and they also appeared in the presence of the filter paper. They were instead due to Marangoni stresses caused by gradients of surface tension at the interface when it was reached by the second monomer. In fact, due to diffusion, different amounts of sebacoyldichloride reached the interface in different places and at different times, causing local gradients of concentration and therefore of interfacial tension leading in turn to Marangoni flows [32]. The flows eventually stopped, when the polymerization reaction had formed a sufficiently stiff membrane at the interface. This phenomenon was unavoidable. Changing both the porosity of the filter paper, as well as the concentration and amount of injected sebacoyldichloride, did not lead to any qualitative change and the only differences were in the waiting time before the onset of the flow. Very interestingly, the occurrence of the Marangoni flows and the polymerization did not disrupt significantly the microstructure of the interface. Figure 5b–e show fluorescence micrographs of the interface before and after polymerization, with and without the filter paper, for two monolayer densities. No qualitative differences were visible.

**Figure 6:**
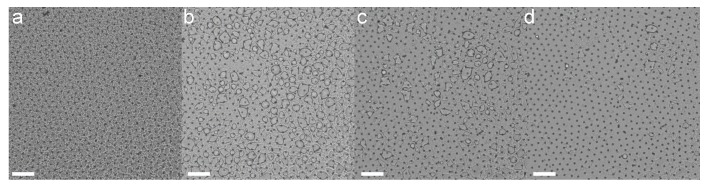
Sequence of bright field microscopy images of the nylon membrane forming at the water–decane interface taken 30 (**a**); 120 (**b**); 180 (**c**) and 240 (**d**) minutes after sebacoyldichloride injection. Scale bars are 50 *μ*m.

This led us to believe that this route yielded a very fast and efficient immobilization mechanism of particle monolayers into polymeric membranes, but additional inspection of the nylon membranes by bright-field microscopy, as reported in Figure 6, showed instead that the completion of the polymerization reaction required significantly longer times. Visible droplets were in fact formed onto the membrane a few minutes after particle immobilization and disappeared over the course of several hours. These were most likely sebacoyldichloride pockets that accumulated on the initially formed membrane and that were then slowly consumed by the diaminohexane diffusing through the continuously growing membrane.

### 2.3. UV Interfacial Polymerization of Styrene

Despite its simplicity, the interfacial polymerization of nylon presents some limitations in terms of the control over the final properties of the membrane since the reaction basically proceeds until all the monomers are consumed. We therefore explored a second route where the polymerization reaction can be externally triggered by UV illumination. The reaction scheme we used is depicted schematically in Figure 1c and it is based on the free radical polymerization of styrene. Radical polymerization reactions belong to the family of chain-growth polymerizations and require an initiator to get started. The polymerization continues as long as the active chain end is not terminated by recombination with an other radical or when all the monomer is consumed [33]. The initiation can be triggered by external stimuli, such as UV light as in our case [33]. In order to improve the mechanical stability of the forming polymer film, we also added *p*-divinylbenzene as crosslinker. The different polarities of all the species make these reactions very suitable to be carried out at fluid–fluid interfaces, where the initiator and monomer can be dispersed into immiscible fluids and meet only at the interface, such as in the case of emulsions [34,35].

**Figure 7:**
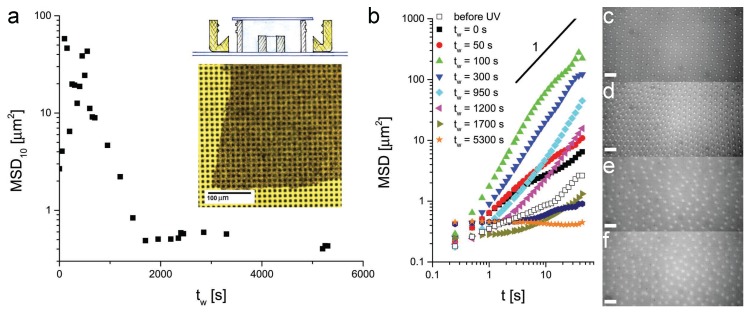
(**a**) MSD calculated after 10 s for 2.8 *μ*m PS particles at a water–decane interface during the interfacial photopolymerization of polystyrene as a function of time after starting the UV illumination $t_{w}$. Top inset: schematics of the sample cell. Bottom inset: micrograph of a composite PS particle-polystyrene membrane deposited onto a pitted silicon wafer with 5×5 *μ*m${}^{2}$ cavities; (**b**) MSD versus time for different representative times during the photopolymerization; (**c–f**) fluorescence images of the 2.8 *μ*m PS particles at the interface before UV illumination (**c**) and after 50 s (**d**), 40 (**e**) and 90 (**f**) minutes. Scale bar: 25 *μ*m.

**Figure 8:**
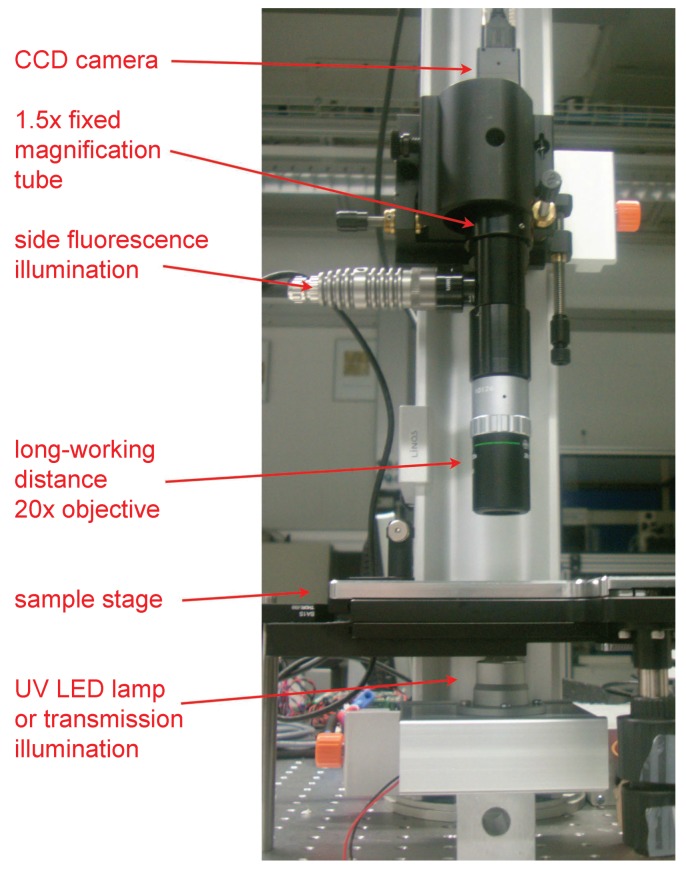
Image and description of the custom microscopy setup.

In the specific case of our experiments, we had a water-soluble initiator (Irgacure 2959) and decane-soluble monomer and crosslinker (styrene and *p*-divinylbenzene). While details of the preparation of the two solutions will be given in the Experimental Section, we report here the essential steps for the particle immobilization experiments. We used a slightly modified version of the sample cell used for the nylon polymerization as sketched in the top inset to Figure 7a. Here, a thicker metal ring was used to form the interface between 112 *μ*L of a 4.46 mM water solution of the initiator and 2 mL of decane containing 1.56 mM of styrene and 0.14 mM of *p*-divinylbenzene. The particles were injected at the interface as previously described. After particle injection, the top part of the cell was completely filled with the decane solution and the cell was carefully sealed with a glass slide. Observation of the particles at the interface was carried out with a custom-built microscope as shown in Figure 8, which allowed simultaneous imaging in reflection and sample illumination with a UV LED lamp emitting at 365 nm. Pendant drop tensiometry investigations showed that, at these concentrations, the water–decane interface was readily covered by a stable layer containing monomer, crosslinker and initiator, which are all surface-active species. After equilibration, the UV lamp was turned on and particle mobility was followed by tracking.

Figure 7a shows the evolution of MSD at ten seconds for 2.8 *μ*m PS particles as a function of time after illumination start-up. Representative MSD curves are shown in Figure 7b for various values of $t_{w}$. From these graphs, we can make similar observations as in the case of the nylon polymerization. We observe that, before illumination, the particles showed the standard expected behavior for particles in the interfacial crystals, as reported earlier in Figure 2. When the photopolymerization began, we observed a significant increase of the particle mobility, again due to the presence of concentration gradients of the reacted species at the interface. Upon continuing UV illumination, the polymerization was completed and the particles were fully immobilized in a polystyrene membarne. We point out that membranes could also be formed in the absence of particles, confirming that their presence does not affect the polymerization reaction significantly.

Despite the fact that all the components necessary for the polymerization were already at the interface before the reaction started, as opposed to the nylon polymerization, the UV photopolymerization disrupted entirely the crystalline arrangement of the particles at the interface, as can be seen in the fluorescence micrographs of Figure 7c–e. This was probably due to the fact that, as the polystyrene membrane grew at the interface, it pushed the particles around in a random fashion, distorting completely the pre-existing arrangement of the particles. Partial swelling in styrene, albeit not detectable by optical microscopy, could also be partly responsible for altering the electrostatic interactions between particles during membrane formation. Similar results were observed when using the 1.08 *μ*m PS particles.

Finally, we attempted the deposition of the photo-polymerized composite membrane onto a solid support to investigate its structural and mechanical properties. The bottom inset to Figure 7a shows a flake of such a membrane deposited on a pitted silicon wafer with 5×5 *μ*m${}^{2}$ holes. The membrane was scooped up from the interface onto the substrate. Unfortunately, the deposited membrane was still too fragile to perform atomic-force microscopy (AFM) investigations and tended to rupture over the cavities on the silicon wafer.

## 3. Conclusions

Our experimental results show that there are several open routes for the immobilization of colloidal particles at fluid interfaces. The first option involves the formation of thin elastic films generated by surface-active substances leaking from the particles themselves. Previous work [36] has shown that exposure to organic solvents (and relatively high temperatures) causes plasticization of polystyrene particles, which can even be strongly deformed as a consequence of adsorption at an oil–water interface. Additional work has also shown that the presence of soluble impurities can very strongly affect the wetting behavior of PS ellipsoids at water–decane interfaces, which had to be subjected to rather harsh cleaning conditions in order to eliminate any undesired contamination [37]. Our work instead showed that this process can be used to our advantage to create very stable crystalline arrays of colloids at the interface. The other two approaches that we investigated went one step further and immobilized the particle monolayer within a polymer membrane.

Immobilizing particle monolayers at an interface has proved to be a very successful way to measure *a posteriori* the contact angle of particles at the interface. A wide range of strategies have been proposed to this end [38], including freezing [39] or gelling the water phase [40], growing metallic caps [41] or swelling the particles [42]. A recent method also involved the growth of a thin cyanoacrylate glue layer at the water–air interface, embedding particles that could be later imaged in an scanning electron microscope (SEM) [43]. Our strategies could provide an extension to the latter technique to oil–water interfaces, even though additional studies elucidating the role of membrane growth on the particle position relative to the interface (e.g., by comparison with the methods above) would be required.

On the other hand, composite particle membranes obtained from interfacial assembly have been previously demonstrated [44,45], and we proposed here two conceptually very simple alternative approaches. Interestingly, both approaches showed that the onset of the polymerization reactions coincided with a a strong increase of particle mobility at the interface caused by Marangoni flows. The interplay between polymerization kinetics and the evolution of the microstructure of the interfacial composite membrane becomes then a very interesting direction for future research, where new strategies to reduce or harness Marangoni flows need to be proposed. The next steps to be carried out after this work are to devise suitable strategies to transfer the interfacial membranes onto solid supports to investigate their structure, e.g., thickness or roughness, and mechanical properties as a function of polymerization conditions in a systematic way.

## 4. Experimental Section

### 4.1. Materials

The particles used in the experiments were polystyrene colloids purchased from microParticles GmbH (Berlin, Germany). We used 2.8±0.04 *μ*m diameter green-fluorescent particles and 1.08±0.04 *μ*m diameter red-fluorescent particles. The particles were received as 2.5 *w/v*% water stock suspensions and diluted to 0.75 and 0.5 *w/v*% in 50:50 ultra-pure water:isopropanol mixtures, for the green and red particles, respectively. Isopropanol (99.95%, Fisher Chemicals, Leicestershire, UK), acetone (99.9%, Sigma-Aldrich, St. Louis, MO, USA), 1,6-Diaminohexane (Sigma-Aldrich), sebacoyldichloride (Merck, Darmstadt, Germany) and 2-Hydroxy-4′-(2-Hydroxyethoxy)-2-Methylpropiophenone (Irgacure 2959) (98%, Aldrich Chemistry, St. Louis, MO, USA) were used as received. Decane (99%, ABCR, Karlsruhe, Germany) was purified to remove polar impurities by five consecutive basic alumina column filtrations, using fresh powder for each one. The purified decane showed a stable interfacial tension of 53±0.5 mN/m against water for a minimum of 2 h. Styrene (>99%, TCI, Portland, OR, USA) and *p*-divinylbenzene (80%, Aldrich Chemistry) were each filtered through a basic alumina column to remove the inhibitor. The activated liquids were kept in the fridge until use. Borosilicate glass slides and the pitted silicon wafers (MakroPore-12-70, Smartmembranes, Germany) were cleaned by consecutive ultra-sonication for 10 min in acetone, isopropanol and then ultra-pure water. This was followed by rinsing with ultra-pure water and drying with a nitrogen stream. Shortly before use they were placed in a UV/ozone cleaner for 2 min (UV/Ozone Procleaner Plus, Bioforce Nanosciences, Ames, IA, USA). Finally, monodisperse polystyrene 300 Da (Polyscience Inc., Warrington, PA, US), monodisperse polystyrene 2200 Da (Alfa Aeser, Heysham, UK), pyrromethene (BODIPY *©* 493/503, Invitrogen, Carlsbad, CA, US) and Macrolex RedG (Lanxess, Leverkusen, Germany) were used to prepare the test solutions for UV-visible spectroscopy and pendant drop tensiometry.

The chemicals for the UV photo-polymerization of styrene were prepared as follows. The water phase and the oil phase were prepared separately inside two round-bottom flasks. The initiator Irgacure 2959 was dissolved in ultra-pure water at a concentration of 4.46 mM and stirred for approximately half an hour. Afterwards, remaining particulate was removed by pressing the solution through a syringe filter. The oil-phase was prepared by adding styrene and *p*-divinylbenzene to decane at concentrations of 1.56 mM and of 0.14 mM, respectively. Before use, oxygen was removed by bubbling the two separate solutions with nitrogen for at least one hour. Fresh solutions were prepared before each experiment.

### 4.2. Sample Cells

Sample cells for observation under the microscope were custom-made by cutting 50 mL TPP centrifuge tubes (Sigma-Aldrich) and gluing them onto borosilicate glass slides using the UV-curable adhesive Norland NOA 61 Norland Products, Cranbury, NJ, US. The interface was created and pinned at polished aluminium or galvanized steel rings of thicknesses and diameters in the range of few millimeters. MN 615 (MACHEREY-NAGEL, Duren, Germany) filter paper was used as a physical barrier for the nylon interfacial polymerization and mounted above the interface by screwing the sides of a TPP centrifuge tube onto its cap glued onto the glass slide.

### 4.3. Microscopy Experiments

Microscopy experiments were carried out on the custom-built optical line shown in Figure 8. The setup allowed for simultaneous bright-field or fluorescence imaging and UV illumination by means of a UV LED lamp with a wavelength of 365 nm. The interface was viewed through a 20× infinity-corrected long-working-distance objective with a 20 mm working distance and an additional fixed in-line magnification of 1.5×. Images were captured with a xiQ USB3 CCD camera (Ximea, Munster, Germany) and recorded using the freeware *μ*Manager (Micro-Manager, US). The sample cell was placed on a stage which allowed for illumination of the interface both in reflection (fluorescence) and transmission. Image series of the particles at the interface were acquired at four frames per second at regular intervals to follow the time evolution of the particle dynamics. The images were analyzed using freely available Matlab code [46] based on the standard code by Cocker and Grier [47].

### 4.4. Pendant Drop Tensiometry and UV-Visible Spectroscopy Experiments

The interfacial activity of the various substances described in the Results section was measured by a pendant drop device (DSA100, Krüss GmbH, Hamburg, Germany). Depending on the substances of interest, the interfacial tension of either aqueous droplets in decane or decane droplets in water (with an inverted needle) was measured as a function of time. The accuracy of the measurements is of ±0.5 mN/m. Spectroscopic analysis to prove the presence of polystyrene oligomers and fluorescent dyes released by the PS in decane was carried out with a UV-Visible spectrometer (Jasco V660, Hachioji, Japan) calibrated against pure decane. Each sample was diluted appropriately before the measurements, since only the nature of the released impurities was of interest and no quantitative analysis was carried out.

## References

[1] Ebert F., Keim P., Maret G. (2008). Local crystalline order in a 2D colloidal glass former. Euro. Phys. J. E.

[2] Bohlein T., Mikhael J., Bechinger C. (2012). Observation of kinks and antikinks in colloidal monolayers driven across ordered surfaces. Nat. Mater..

[3] Skinner T.O.E., Aarts D.G.A.L., Dullens R.P.A. (2010). Grain-Boundary Fluctuations in Two-Dimensional Colloidal Crystals. Phys. Rev. Lett..

[4] Isa L., Kumar K., Müller M., Grolig J., Textor M., Reimhult E. (2010). Particle Lithography from Colloidal Self-Assembly at Liquid-Liquid Interfaces. ACS Nano.

[5] Vogel N., Weiss C.K., Landfester K. (2012). From soft to hard: the generation of functional and complex colloidal monolayers for nanolithography. Soft Matter.

[6] Ray M.A., Shewmon N., Bhawalkar S., Jia L., Yang Y.Z., Daniels E.S. (2009). Submicrometer Surface Patterning Using Interfacial Colloidal Particle Self-Assembly. Langmuir.

[7] Binks B.S., Horozov T.S. (2006). Colloidal Particles at Liquid Interfaces.

[8] Kang C., Ramakrishna S.N., Nelson A., Cremmel C.V.M., vom Stein H., Spencer N.D., Isa L., Benetti E.M. (2015). Ultrathin, freestanding, stimuli-responsive, porous membranes from polymer hydrogel-brushes. Nanoscale.

[9] Hanarp P., Sutherland D., Gold J., Kasemo B. (1999). Nanostructured model biomaterial surfaces prepared by colloidal lithography. Nanostruct. Mater..

[10] Hanarp P., Sutherland D.S., Gold J., Kasemo B. (2003). Control of nanoparticle film structure for colloidal lithography. Coll. Surf. A Physicochem. Eng. Asp..

[11] Min W.L., Jiang P., Jiang B. (2008). Large-scale assembly of colloidal nanoparticles and fabrication of periodic subwavelength structures. Nanotechnology.

[12] Denkov N.D., Velev O.D., Kralchevsky P.A., Ivanov I.B., Yoshimura H., Nagayama K. (1992). Mechanism of formation of 2-dimensional crystals from latex-particles on substrates. Langmuir.

[13] Gu Z.Z., Fujishima A., Sato O. (2002). Fabrication of High-Quality Opal Films with Controllable Thickness. Chem. Mater..

[14] Zhang K.Q., Liu X.Y. (2004). In situ observation of colloidal monolayer nucleation driven by an alternating electric field. Nature.

[15] Velev O., Bhatt K.H. (2006). On-chip micromanipulation and assembly of colloidal particles by electric fields. Soft Matter.

[16] Reimhult E., Kumar K. (2008). Membrane biosensor platforms using nano- and microporous supports. Trends Biotechnol..

[17] Elnathan R., Isa L., Brodoceanu D., Nelson A., Harding F.J., Delalat B., Kraus T., Voelcker N.H. (2015). Versatile Particle-Based Route to Engineer Vertically Aligned Silicon Nanowire Arrays and Nanoscale Pores. ACS Appl. Mater. Interfaces.

[18] Rey B.M., Elnathan R., Ditcovski R., Geisel K., Zanini M., Fernandez-Rodriguez M.A., Naik V.V., Frutiger A., Richtering W., Ellenbogen T. (2016). Fully Tunable Silicon Nanowire Arrays Fabricated by Soft Nanoparticle Templating. Nano Lett..

[19] Ramakrishna S.N., Clasohm L.Y., Rao A., Spencer N.D. (2011). Controlling Adhesion Force by Means of Nanoscale Surface Roughness. Langmuir.

[20] Ramakrishna S.N., Nalam P.C., Clasohm L.Y., Spencer N.D. (2013). Study of Adhesion and Friction Properties on a Nanoparticle Gradient Surface: Transition from JKR to DMT Contact Mechanics. Langmuir.

[21] Vogel N., Goerres S., Landfester K., Weiss C.K. (2011). A Convenient Method to Produce Close- and Non-close-Packed Monolayers using Direct Assembly at the Air–Water Interface and Subsequent Plasma-Induced Size Reduction. Macromol. Chem. Phys..

[22] Li X., Wang T., Zhang J., Yan X., Zhang X., Zhu D., Li W., Zhang X., Yang B. (2010). Modulating Two-Dimensional Non-Close-Packed Colloidal Crystal Arrays by Deformable Soft Lithography. Langmuir.

[23] Ray M.A., Jia L. (2007). Micropatterning by non-densely packed interfacial colloidal crystals. Adv. Mater..

[24] Pieranski P. (1980). Two-dimesional interfacial colloidal crystals. Phys. Rev. Lett..

[25] Horozov T.S., Aveyard R., Clint J.H., Binks B.P. (2003). Order-Disorder Transition in Monolayers of Modified Monodisperse Silica Particles at the Octane-Water Interface. Langmuir.

[26] Petkov P.V., Danov K.D., Kralchesvky P.A. (2014). Surface Pressure Isotherm for a Monolayer of Charged Colloidal Particles at a Water/Nonpolar–Fluid Interface: Experiment and Theoretical Model. Langmuir.

[27] Petkov P.V., Danov K.D., Kralchesvky P.A. (2016). Monolayers of charged particles in a Langmuir trough: Could particle aggregation increase the surface pressure?. J. Coll. Interface Sci..

[28] Bonales L.J., Rubio J.E.F., Ritacco H., Vega C., Rubio R.G., Ortega F. (2011). Freezing Transition and Interaction Potential in Monolayers of Microparticles at Fluid Interfaces. Langmuir.

[29] Rey M., Fernandez-Rodriguez M.A., Steinacher M., Scheidegger L., Geisel K., Richtering W., Squires T.M., Isa L. (2016). Isostructural solid-solid phase transition in monolayers of soft core-shell particles at fluid interfaces: Structure and mechanics. Soft Matter.

[30] Reynaert S., Moldenaers P., Vermant J. (2006). Control over colloidal aggregation in monolayers of latex particles at the oil-water interface. Langmuir.

[31] Morgan P.W., Kwolek S.L. (1959). The nylon rope trick: Demonstration of condensation polymerization. J. Chem. Educ..

[32] Barnes G.T., Gentle I.R. (2011). Interfacial Science: An Introduction.

[33] Kaiser W. (2011). Kunststoffchemie Für Ingenieure.

[34] Hunkeler D., Candau F., Pichot C., Hemielec A.E., Xie T.Y., Barton J., Vaskova V., Guillot J., Dimonie M.V., Reichert K.H. (1994). Heterophase polymerizations: A physical and kinetic comparison and categorization. Theories and Mechanism of Phase Transitions, Heterophase Polymerizations, Homopolymerization, Addition Polymerization.

[35] Cunningham M.F. (2002). Living/controlled radical polymerizations in dispersed phase systems. Prog. Polym. Sci..

[36] Park B.J., Furst E.M. (2010). Fabrication of Unusual Asymmetric Colloids at an Oil–Water Interface. Langmuir.

[37] Coertjens S., Moldenaers P., Vermant J., Isa L. (2014). Contact Angles of Microellipsoids at Fluid Interfaces. Langmuir.

[38] Zanini M., Isa L. (2016). Particle contact angles at fluid interfaces: Pushing the boundary beyond hard uniform spherical colloids. J. Phys. Condens. Matter.

[39] Isa L., Lucas F., Wepf R., Reimhult E. (2011). Measuring single-nanoparticle wetting properties by freeze-fracture shadow-casting cryo-scanning electron microscopy. Nat. Commun..

[40] Paunov V.N. (2003). Novel Method for Determining the Three-Phase Contact Angle of Colloid Particles Adsorbed at Air–Water and Oil–Water Interfaces. Langmuir.

[41] Sabapathy M., Kollabattula V., Basavaraj M.G., Mani E. (2015). Visualization of the equilibrium position of colloidal particles at fluid–water interfaces by deposition of nanoparticles. Nanoscale.

[42] Lu Z., Zhou M. (2011). Fabrication of large scale two-dimensional colloidal crystal of polystyrene particles by an interfacial self-ordering process. J. Coll. Interface Sci..

[43] Vogel N., Ally J., Bley K., Kappl M., Landfester K., Weiss C.K. (2014). Direct visualization of the interfacial position of colloidal particles and their assemblies. Nanoscale.

[44] Kiesow I., Marczewski D., Reinhardt L., Mühlmann M., Possiwan M., Goedel W.A. (2013). Bicontinuous Zeolite Polymer Composite Membranes Prepared via Float Casting. J. Am. Chem. Soc..

[45] Xu H., Goedel W.A. (2003). From Particle-Assisted Wetting to Thin Free-Standing Porous Membranes. Angew. Chem. Int. Ed..

[46] MATLAB. http://people.umass.edu/kilfoil/downloads.html.

[47] Crocker J.C., Grier D.G. (1996). Methods of digital video microscopy for colloidal studies. J. Coll. Interface Sci..

